# Predictive Factors for a Satisfactory Treatment Outcome with Intravesical Botulinum Toxin A Injection in Patients with Interstitial Cystitis/Bladder Pain Syndrome

**DOI:** 10.3390/toxins11110676

**Published:** 2019-11-19

**Authors:** Hsiu-Jen Wang, Wan-Ru Yu, Hueih-Ling Ong, Hann-Chorng Kuo

**Affiliations:** 1 Department of Urology, Hualien Tzu Chi Hospital, Buddhist Tzu Chi Medical Foundation and Tzu Chi University, Hualien 970, Taiwan; 2 Department of Nursing, Hualien Tzu Chi Hospital, Buddhist Tzu Chi Medical Foundation and Tzu Chi University, Hualien 970, Taiwan; 3 Department of Urology, Dalin Tzu Chi Hospital, Buddhist Tzu Chi Medical Foundation, Chiayi 622, Taiwan

**Keywords:** bladder pain, botulinum toxin A, predictor, maximal bladder capacity, hydrodistention

## Abstract

A botulinum toxin A (BoNT-A) intravesical injection can improve the symptoms of interstitial cystitis/bladder pain syndrome (IC/BPS). Patients with IC/BPS have different clinical characteristics, urodynamic features, and cystoscopic findings. This study assessed the treatment outcomes of a BoNT-A intravesical injection and aimed to identify the predictive factors of a satisfactory outcome. This retrospective study included IC/BPS patients treated with 100 U BoNT-A. The treatment outcomes were assessed by global response assessment (GRA) at 6 months. We classified patients according to different clinical, urodynamic, and cystoscopic characteristics and evaluated the treatment outcomes and predictive factors. A total of 238 patients were included. Among these patients, 113 (47.5%) had a satisfactory outcome (GRA ≥ 2) and 125 (52.5%) had an unsatisfactory outcome. Improvements in the IC symptom score, IC problem score, O’Leary–Sant symptom score, and visual analog scale score for pain were significantly greater in patients with a satisfactory outcome than in patients with an unsatisfactory outcome (all *p* = 0.000). The IC disease duration and maximal bladder capacity (MBC) were significantly different between patients with and without a satisfactory outcome. Multivariate analysis revealed that only the MBC was a predictor for a satisfactory outcome. Patients with a MBC of ≥760 mL and glomerulations of 0/1 (58.7%) or glomerulations of 2/3 (75.0%) frequently had a satisfactory outcome. We found that BoNT-A intravesical injection can effectively improve symptoms among patients with IC/BPS, with a remarkable reduction in bladder pain. A MBC of ≥760 mL is a predictive factor for a satisfactory treatment outcome.

## 1. Introduction

Interstitial cystitis/bladder pain syndrome (IC/BPS) is a clinical symptom syndrome characterized by frequency, urgency, and nocturia, with or without bladder pain [1]. Although this mysterious disease has been documented for over 100 years, the underlying pathophysiology is still not well elucidated. IC/BPS is usually classified as ulcer type (with Hunner’s lesion) or non-ulcer type (with glomerulations after cystoscopic hydrodistention) [1,2]. Among all symptoms, pelvic pain has been considered as the primary symptom of IC/BPS [3]. However, a previous study found that some patients presenting with similar indicative symptoms did not show remarkable glomerulations, and a hypersensitive bladder was considered [4].

Chronic inflammation of the bladder has been accepted as the fundamental cause of IC/BPS [5]; however, the reasons for inflammation are unclear. This unsolved bladder inflammation results in denudation of the urothelium, abnormal expression of tight junction proteins, deficiency of barrier proteins, altered differentiation of urothelial cells, and increased permeability of the bladder urothelium [6,7,8,9,10,11]. A previous study demonstrated that intravesical botulinum toxin A (BoNT-A) can reduce bladder pain in IC/BPS patients [12]. Additionally, repeated BoNT-A intravesical injections have been shown to increase functional bladder capacity and reduce bladder pain in responders [13]. In IC/BPS patients, BoNT-A treatment could gradually reduce bladder inflammation and improve urothelial repair, leading to symptomatic relief [14].

Clinically, not all IC/BPS patients have the same bladder characteristics and histological findings. Some IC/BPS patients have comorbid systemic diseases and mental illnesses [14,15]. Functional somatic syndrome has been found to be closely associated with IC/BPS [16]. IC/BPS might involve heterogeneous subgroups of patients who present with different clinical, urodynamic, and cystoscopic characteristics [17].

Although BoNT-A intravesical injection is listed as the third-line therapeutic option for IC/BPS, the treatment effect has not been well demonstrated in real-world practice, and the predictors for a satisfactory outcome have not been clarified. A previous study on BoNT-A injection for IC/BPS revealed excellent symptom improvement in approximately 60% of patients [18]. However, the long-term treatment efficacy of BoNT-A injections with regard to IC/BPS has not been established [19], and repeat intravesical BoNT-A injections are necessary to achieve a better long-term outcome [20]. The treatment efficacy of BoNT-A injections might differ depending on the IC/BPS phenotype. 

The present study aimed to assess the treatment outcomes of an intravesical BoNT-A injection and identify the predictive factors for a satisfactory treatment outcome in patients with IC/BPS. 

## 2. Results

A total of 238 patients (38 male and 200 female patients) were included in this study. The mean patient age at the diagnosis of IC/BPS was 40.2 ± 13.5 years, and the mean duration of their treatment course for IC/BPS was 15.1 ± 9.5 years. According to the definition of treatment outcome, a satisfactory outcome (global response assessment (GRA) ≥ 2) was noted in 113 (47.5%) patients and an unsatisfactory outcome (GRA ≤ 1) was noted in 125 (52.5%) patients. In the beginning, we used receiver operation characteristics to analyze the cut-off value for a satisfactory treatment outcome. According to the treatment outcome, the result revealed that a maximal bladder capacity (MBC) of ≥760 mL had the greatest area under the curve (AUC = 0.547). Patients with a satisfactory outcome had a significantly greater MBC than those with an unsatisfactory outcome (684.5 ± 197.5 mL vs. 619.1 ± 192.3 mL, *p* = 0.010). The clinical characteristics, urodynamic findings, and cystoscopic findings of the satisfactory and unsatisfactory groups are listed in Table 1. The IC/BPS duration was significantly shorter and the rate of MBC ≥ 760 mL was significantly higher in the satisfactory group than in the unsatisfactory group (*p* = 0.017 and *p* = 0.002, respectively). The other variables showed no significant differences between the groups. Therefore, we further classified the patients into five subgroups according to the glomerulation grade (0/1, 2/3, and Hunner’s lesion) and MBC (≥760 mL or <760 mL) during cystoscopic hydrodistention. Patients with glomerulation grade 2/3 and MBC ≥ 760 mL had a satisfactory rate of 75%, and those with glomerulation grade 0/1 and MBC ≥ 760 mL had a satisfactory rate of 58.7%, the satisfactory rates of the other subgroups were approximately 40% (*p* = 0.024) (Table 2). Multivariate analysis revealed that a MBC ≥ 760 mL (*p* = 0.000) and the IC/BPS phenotype (*p* = 0.012) were predictive factors for a satisfactory outcome.

The changes in clinical symptoms and the urodynamic parameters after BoNT-A injections are shown in Table 3. Both the satisfactory and unsatisfactory groups showed improvements in the interstitial cystitis symptom index (ICSI), interstitial cystitis problem index (ICPI), O’Leary–Sant symptom score (OSS), and visual analog scale (VAS) score after BoNT-A treatment. However, the improvements in symptomatic variables were significantly greater in the satisfactory group than those in the unsatisfactory group. The urodynamic parameters revealed that both the satisfactory and unsatisfactory groups had an increase in the bladder capacity of first sensation of bladder filling (FSF) and post-void residual (PVR) volume, while the voided volume did not change; however, a significant change in the detrusor pressure after a BoNT-A intravesical injection was noted only in the patients with a satisfactory outcome.

Adverse events were hematuria after injection in 12 (5%) patients, urinary tract infection in 18 (7.6%), and mild dysuria in 24 (10.1%). No urinary retention was reported in any of the patients treated. 

## 3. Discussion

The results of this study demonstrated that an intravesical BoNT-A injection greatly improved symptoms in 47.5% of IC/BPS patients with a long disease duration and refractory to medical treatment. Patients with an unsatisfactory outcome also showed symptomatic improvement, but the urodynamic parameters did not change after treatment. A MBC ≥ 760 mL during cystoscopic hydrodistention predicts a satisfactory outcome, regardless of the glomerulation grade.

A BoNT-A intravesical injection for the treatment of IC/BPS has been attempted for the last 15 years. An intravesical BoNT-A (100–200 U) injection into the detrusor plus hydrodistention has been shown to be effective for alleviating bladder pain and decreasing frequency nocturia in IC/BPS patients [21]. Additionally, a previous randomized, double-blind clinical trial demonstrated that BoNT-A (100 U) is effective and safe for the treatment of IC/BPS [18]. The bladder pain reduction and bladder capacity improvement were significantly greater in the patients receiving BoNT-A treatment than in the placebo group [18]. In this study, we only found that a single BoNT-A injection increased the FSF, but not the voided volume. A BoNT-A injection in IC/BPS bladders has been found to inhibit noxious neurotransmitter releases, including calcitonin gene-related peptide, glutamate, adenosine triphosphate, and substance P from neurons [22]. Therefore, the therapeutic effect of BoNT-A on sensory afferents could reduce the sensory urgency and increase FSF. With solid evidence, an intravesical BoNT-A injection has been listed as a fourth-line therapeutic option in the clinical guidelines for IC/BPS in the American Urological Association [23].

The clinical presentations of IC/PBS are frequency and urgency with or without bladder and lower abdominal pain, glomerulations developed after cystoscopic hydrodistention, and denudation of the bladder urothelium [5]. As the actual pathophysiology of IC/BPS has not been well elucidated, there could be different underlying pathologies in IC/BPS bladders, resulting in heterogeneous clinical phenotypes. Among the different clinical characteristics of IC/BPS, Hunner’s lesion, bladder pain symptoms, glomerulation grade, and MBC after cystoscopic hydrodistention might imply different pathophysiologies in the diseased bladder. A bladder with a Hunner’s lesion usually has chronic inflammation at the lesion, and it causes severe pain when the bladder is distended. High expressions of T-cell and B-cell makers in the submucosa and high urinary immunoglobulin levels have been noted in Hunner’s lesion [24]. Urothelial dysfunction due to abnormal urothelial cell differentiation, loss of E-cadherin and zonula occluden-1 expression, and deficient differentiation makers are remarkable in IC/BPS bladders [24]. High acute and chronic bladder inflammation, high lymphocyte infiltration, but not mast cell activation, limit the bladder capacity during cystoscopic hydrodistention and cause a low anesthetic bladder capacity [25]. High sensory afferent nerve activity causes an increase in bladder pain and a small functional capacity in daily life [26].

The previous classification usually divided IC/BPS into Hunner’s lesion and non-ulcer IC/BPS. This classification was based on histopathologic findings, distinct clinical characteristics, and underlying pathophysiology [27]. Recent Asian IC guidelines added the hypersensitive bladder subtype to define a subgroup of patients with IC clinical symptoms but no glomerulation [4]. In real-world practice, we noted that the glomerulation grade and MBC might have different combinations during cystoscopic hydrodistention. In this study, a MBC ≥ 760 mL was found to have a significantly better treatment outcome after the intravesical BoNT-A injection. The glomerulation grade does not have a predictive value for the BoNT-A treatment outcome. If glomerulation and MBC are combined for IC/BPS, a satisfactory treatment outcome is still mainly based on a MBC ≥ 760 mL, suggesting that a larger MBC indicates a better response to a BoNT-A injection.

BoNT-A has been demonstrated to have anti-inflammatory effects on the chronic cystitis rat model and to reduce the expression of nerve growth factors in IC/BPS bladders, with satisfactory pain relief [28,29]. In addition, a BoNT-A injection relaxes the smooth muscles and increases functional bladder capacity through the inhibition of acetylcholine release. After a BoNT-A injection, both motor and sensory nerves are affected and patients experience symptomatic relief [18]. The results of this study demonstrate that patients with a satisfactory outcome showed a great decrease in detrusor pressure after the BoNT-A intravesical injection, suggesting a strong response of the detrusor muscle to the BoNT-A injection in patients with a satisfactory outcome. Although the PVR volume increased significantly after the BoNT-A injection in both patients with a satisfactory outcome and those with an unsatisfactory outcome, there was no significant difference between group. The increased PVR was not clinically significant, because only 10.1% of patients complained of dysuria after the BoNT-A injection. Moreover, no patient reported acute urinary retention after the BoNT-A injection, indicating that BoNT-A is a safe and effective treatment for IC/BPS refractory to lifestyle modification and medical treatment. Although the satisfactory rate is not high after one single BoNT-A injection, our previous study showed that repeated BoNT-A injections could improve the satisfactory rate [20].

## 4. Conclusions

The present study showed that a BoNT-A injection is effective for pain relief and symptom improvement among IC/BPS patients. The improvement in bladder pain is remarkable in patients with a satisfactory treatment outcome. A MBC ≥ 760 mL is a predictive factor for a satisfactory treatment outcome, whereas glomerulation grade and urodynamic parameters do not have predictive value for the IC/BPS treatment outcome.

## 5. Materials and Methods

This retrospective study included IC/BPS patients who had been treated for the first time with 100 U of BoNT-A (BOTOX, Allergan, Irvine, CA, USA) in 10 mL saline injected at 20 sites. All patients had been diagnosed to have IC/BPS based on the characteristic IC symptoms and cystoscopic findings of glomerulations, petechiae, or mucosal fissures on hydrodistention under anesthesia [30]. Among the IC/BPS patients, at least two treatment modalities had been tried, including lifestyle modification, cystoscopic hydrodistention, intravesical hyaluronic acid instillation, or painkiller medication, but the IC symptoms were persistent or had relapsed.

All patients received a thorough investigation at enrollment and were not included if they failed to meet the inclusion criteria of the National Institute of Diabetes and Digestive and Kidney Diseases (NIDDK) [31]. The treatment outcomes were assessed by the global response assessment (GRA) at 6 months after an intravesical BoNT-A injection. In addition, the IC symptoms were assessed by the O’Leary–Sant symptom score (OSS), including the IC symptom index (ICSI) and IC problem index (ICPI) [32]. The bladder pain was also reported by a self-assessed 10-point visual analog scale (VAS).

This study had been approved by the Research Ethics Committee of Hualien Tzu Chi Hospital, Buddhist Tzu Chi Medical Foundation (IRB 105-25-B, date of approval: 2 June 2018). The written informed consent was waived due to the nature of retrospective analysis.

A videourodynamic study (VUDS) was performed before the BoNT-A injection, using standard procedures. The VUDS involved the infusion of normal saline at a rate of 20 mL/min. All descriptions and terminologies used in this study were according to the recommendations of the International Continence Society [33]. The reported urodynamic parameters included: FSF, urge sensation, cystometric bladder capacity, pressure, maximum flow rate (Qmax) and detrusor pressure at Qmax during voiding, and post-void residual (PVR) volume. According to the characteristic VUDS findings, the urodynamic diagnosis was recorded as increased bladder sensation, detrusor overactivity, dysfunctional voiding, poor pelvic floor muscle relaxation, or intrinsic sphincter deficiency [34]. The potassium chloride (KCl) test (infusion of 0.4 M KCl in normal saline) was performed after evacuation of the PVR urine. The KCl test was considered positive if there was bladder pain or a severe urge to void during the KCl infusion [35]. The VUDS was performed at baseline to confirm the diagnosis of IC/BPS and search for other bladder conditions mimicking IC/BPS. A repeat VUDS was performed at 6 months after the first BoNT-A injection to evaluate the bladder condition after treatment and as a guide for the selection of the next step treatment.

The injected BoNT-A solution constituted a vial of onabotulinumtoxinA (100 U) diluted with 10 mL of normal saline. Each 1.0 mL solution contained 10 U of BoNT-A in saline. A total of 20 injections were performed with this BoNT-A solution, resulting in 5 U of BoNT-A in each injection site. The injection technique was reported in our previous article [18]. In brief, the BoNT-A solution was injected in four rows (each row with 5 injection sites) from the interureteric ridge to the bladder dome to cover the lateral and posterior wall, at a bladder capacity of 100 mL. The trigone was spared, because glomerulations are usually not involved in this region. After the BoNT-A injections, cystoscopic hydrodistention was routinely performed by a slow infusion of normal saline to an 80 cm H_2_O intravesical pressure for 15 min. The maximal bladder capacity (MBC) at the end of the infusion and the degree of glomerulations developed after bladder drainage were also recorded [18]. The glomerulation grade was classified as 0, 1, 2, and 3 based on the appearance of glomerulations at none, less than half, more than half, and more than half of the bladder wall and severe waterfall bleeding, respectively. Patients were classified as ulcer type IC/BPS if a Hunner’s lesion was present with and without glomerulations [4]. After BoNT-A treatment, patients were indwelled with a 14-Fr Foley catheter overnight and discharged the next day. An antibiotic (cephalexin 500 mg every 6 h) was routinely prescribed for seven days and patients visited the outpatient clinic at 2, 4, and 8 weeks after treatment, followed by monthly visits in the outpatient clinic for outcome assessment. The primary end-point was 6 months after the BoNT-A injection.

We classified patients into five subgroups according to different clinical, urodynamic, and cystoscopic characteristics (MBC and glomerulations) and evaluated the treatment outcome (GRA ≥ 2 or GRA ≤ 1), symptom change (with and without bladder pain), urodynamic findings, and comorbidity coexistence. Predictive factors for a satisfactory outcome were also assessed.

The continuous variables reported in this study are expressed as means ± standard deviations (SDs). The Wilcoxon rank-sum test was used for statistical comparisons of continuous variables between groups, and the Wilcoxon sign-rank test was used to evaluate the difference of the variables between baseline and post-treatment. All statistical assessments were performed by two-sided analysis, and significant differences were considered at a *p*-value < 0.05. SPSS version 15.0 statistical software (SPSS Inc., Chicago, IL, USA) was used in all statistical analyses. 

## Figures and Tables

**Table 1:** Characteristics of the study patients according to the treatment outcome.

Characteristics	Item	Unsatisfactory Outcome GRA ≤ 1 (n = 125)	Satisfactory Outcome GRA ≥ 2 (n = 113)	Univariate *p*-Value	Multivariate *p*-Value
Sex (male:female)		18:107	20:93	0.488	
Age at IC symptom (years)		39.8 ± 13.6	42.7 ± 13.7	0.127	
IC duration (years)		16.6 ± 10.7	13.5 ± 8.64	0.017	
Comorbidity	≥2 ≤1	64 (52.0%) 61 (55.7%)	59 (48.0%) 54 (44.3%)	0.532	
Bladder pain	Yes no	86 (56.2%) 39 (45.9%)	67 (43.8%) 46 (54.1%)	0.126	
Increased bladder sensation	Yes No	107 (52.2%) 18 (50.5%)	98 (47.8%) 15 (45.5%)	0.802	0.292
Detrusor overactivity	Yes No	16 (53.3%) 109 (52.4%)	14 (46.7%) 99 (47.6%)	0.924	0.904
Bladder neck dysfunction	Yes No	4 (40.4%) 121 (53.1%)	6 (60.0%) 107 (46.9%)	0.524	0.732
Dysfunctional voiding	Yes No	9 (52.9%) 116 (52.5%)	8 (47.1%) 105 (47.5%)	0.971	0.619
Poor PFM relaxation	Yes No	62 (56.9%) 63 (48.8%)	47 (43.1%) 66 (51.2%)	0.216	0.206
Intrinsic sphincter deficiency	Yes No	4 (66.7%) 121 (52.2%)	2 (33.3%) 111 (47.8%)	0.686	0.087
Maximal bladder capacity (mL)	Mean ≥760 <760	619.1 ± 192.3 24 (36.4%) 101 (58.7%)	684.5 ± 197.5 42 (63.6%) 71 (41.3%)	0.010 0.002	0.000
Glomerulation	0/1 2/3 ulcer	47 (51.1%) 71 (53.4%) 7 (53.8%)	45 (48.9%) 62 (46.6%) 6 (46.2%)	0.940	0.537
IC phenotype		125 (52.5%)	113 (47.5%)	0.024	0.012

GRA, global response assessment; IC, interstitial cystitis; PFM, pelvic floor muscle.

**Table 2:** Cystoscopic phenotype distribution according to the treatment outcome.

Phenotype	Unsatisfactory Outcome GRA ≤ 1	Satisfactory Outcome GRA ≥ 2	Total
Glomerulation 0/1, MBC ≥ 760 mL	19 (41.3%)	27 (58.7%)	46 (19.3%)
Glomerulation 0/1, MBC < 760 mL	28 (60.9%)	18 (39.1%)	46 (19.3%)
Glomerulation 2/3, MBC ≥ 760 mL	5 (25.0%)	15 (75.0%)	20 (8.4%)
Glomerulation 2/3, MBC < 760 mL	66 (58.4%)	47 (41.6%)	113 (47.5%)
With Hunner’s lesion	7 (53.8%)	6 (46.2%)	13 (5.5%)
Total	125 (52.5%)	113 (47.5%)	238 (100%)

GRA, global response assessment; MBC, maximal bladder capacity.

**Table 3:** Changes in symptom scores and urodynamic parameters from baseline to 6 months after intravesical Botox injection according to the treatment outcome.

Urodynamic Parameters	Time Point	Unsatisfactory Outcome GRA ≤ 1 (n = 125)	Satisfactory Outcome GRA ≥ 2 (n = 113)	Total (n = 238)
ICSI	BL FU	12.2 ± 3.70 9.41 ± 4.71 *	12.6 ± 3.77 4.97 ± 3.88 *#	
ICPI	BL FU	11.5 ± 3.07 9.45 ± 4.41 *	12.0 ± 3.39 4.59 ± 4.23 *#	
OSS	BL FU	23.7 ± 6.39 18.9 ± 8.56 *	24.6 ± 6.64 9.56 ± 7.77 *#	
VAS score	BL FU	4.48 ± 32.42 3.87 ± 3.38	5.19 ± 2.77 2.15 ± 2.74 *#	
First sensation (mL)	BL FU	112 ± 50.5 126 ± 67.9 *	117 ± 51.9 130 ± 58.2 *	115 ± 51.2 128 ± 63.2 *
Full sensation (mL)	BL FU	177 ± 74.2 190 ± 91.1	184 ± 73.3 205 ± 90.0	180 ± 7.7 197 ± 90.6 *
Urge sensation (mL)	BL FU	216 ± 86.6 222 ± 110	229 ± 90.1 238 ± 110	222 ± 88.4 230 ± 110
Detrusor pressure (cmH_2_O)	BL FU	20.4 ± 12.7 21.4 ± 25.7	21.9 ± 14.9 18.2 ± 14.4 *	21.1 ± 13. 819.9 ± 20.9
Maximum flow rate (mL/s)	BL FU	12.0 ± 6.51 10.5 ± 5.97	12.5 ± 4.94 12.4 ± 6.30	12.3 ± 5.79 11.4 ± 6.19
Voided volume (mL)	BL FU	232 ± 113 227 ± 139	268 ± 130 253 ± 131	249 ± 123 240 ± 135
Post-void residual volume (mL)	BL FU	39.4 ± 71.3 70.4 ± 106 *	26.9 ± 53.4 48.0 ± 82.1 *	33.2 ± 63.3 59.3 ± 95.2 *
Cystometric bladder capacity (mL)	BL FU	273 ± 109 290 ± 147	297 ± 126 304 ± 126	285 ± 11 8297 ± 137
Bladder compliance (mL/cmH_2_O)	BL FU	63.4 ± 67.0 62.7 ± 60.0	60.0 ± 61. 879.7 ± 88.5	61.7 ± 64.4 71.1 ± 75.7

* *p* < 0.05 in variables between baseline and follow-up within group; # *p* < 0.05 in the change of variables from baseline to follow-up between group. GRA, global response assessment; ICSI, interstitial cystitis symptom index; ICPI, interstitial cystitis problem index; OSS, O’Leary–Sant symptom score; VAS, visual analog scale; BL, baseline; FU, follow-up.

## References

[1] Bouchelouche K., Nordling J. (2003). Recent developments in the management of interstitial cystitis. Curr. Opin. Urol..

[2] Hanno P.M., Sant G.R. (2001). Clinical highlights of the National Institute of Diabetes and Digestive and Kidney Diseases/Interstitial Cystitis Association scientific conference on interstitial cystitis. Urology.

[3] Hanno P.M., Burks D.A., Clemens J.Q., Dmochowski R.R., Erickson D., Fitzgerald M.P., Forrest J.B., Gordon B., Gray M., Mayer R.D. (2011). AUA guideline for the diagnosis and treatment of interstitial cystitis/bladder pain syndrome. J. Urol..

[4] Homma Y., Ueda T., Tomoe H., Lin A.T., Kuo H.C., Lee M.H., Oh S.J., Kim J.C., Lee K.S. (2016). Clinical guidelines for interstitial cystitis and hypersensitive bladder updated in 2015. Int. J. Urol..

[5] Sant G.R., Kempuraj D., Marchand J.E., Theoharides T.C. (2007). The mast cell in interstitial cystitis: Role in pathophysiology and pathogenesis. Urology.

[6] Shie J.H., Kuo H.C. (2011). Higher levels of cell apoptosis and abnormal E-cadherin expression in the urothelium are associated with inflammation in patients with interstitial cystitis/painful bladder syndrome. BJU Int..

[7] Southgate J., Varley C.L., Garthwaite M.A., Hinley J., Marsh F., Stahlschmidt J., Trejdosiewicz L.K., Eardley I. (2007). Differentiation potential of urothelium from patients with benign bladder dysfunction. BJU Int..

[8] Zeng Y., Wu X.X., Homma Y., Yoshimura N., Iwaki H., Kageyama S., Yoshiki T., Kakehi Y. (2007). Uroplakin III-delta4 messenger RNA as a promising marker to identify nonulcerative interstitial cystitis. J. Urol..

[9] Hauser P.J., Dozmorov M.G., Bane B.L., Slobodov G., Culkin D.J., Hurst R.E. (2008). Abnormal expression of differentiation related proteins and proteoglycan core proteins in the urothelium of patients with interstitial cystitis. J. Urol..

[10] Kim J., Keay S.K., Dimitrakov J.D., Freeman M.R. (2007). p53 mediates interstitial cystitis antiproliferative factor (APF)-induced growth inhibition of human urothelial cells. FEBS Lett..

[11] Parsons C.L. (2011). The role of a leaky epithelium and potassium in the generation of bladder symptoms in interstitial cystitis/overactive bladder, urethral syndrome, prostatitis and gynaecological chronic pelvic pain. BJU Int..

[12] Lee C.L., Kuo H.C. (2015). Long-term efficacy and safety of repeated intravescial onabotulinumtoxinA injections plus hydrodistention in the treatment of interstitial cystitis/ bladder pain syndrome. Toxins.

[13] Shie J.H., Liu H.T., Wang Y.S., Kuo H.C. (2013). Immunohistochemical evidence suggests repeated intravesical application of botulinum toxin A injections may improve treatment efficacy of interstitial cystitis/bladder pain syndrome. BJU Int..

[14] Keller J.J., Chen Y.K., Lin H.C. (2012). Comorbidities of bladder pain syndrome/interstitial cystitis: A population-based study. BJU Int..

[15] Nickel J.C., Tripp D.A., International Interstitial Cystitis Study Group (2015). Clinical and psychological parameters associated with pain pattern phenotypes in women with interstitial cystitis/bladder pain syndrome. J. Urol..

[16] Clemens J.Q., Elliott M.N., Suttorp M., Berry S.H. (2012). Temporal ordering of interstitial cystitis/bladder pain syndrome and non-bladder conditions. Urology.

[17] Fuoco M.B., Irvine-Bird K., Curtis Nickel J. (2014). Multiple sensitivity phenotype in interstitial cystitis/bladder pain syndrome. Can. Urol. Assoc. J..

[18] Kuo H.C., Jiang Y.H., Tsai Y.C., Kuo Y.C. (2016). Intravesical botulinum toxin-A injections reduce bladder pain of interstitial cystitis/bladder pain syndrome refractory to conventional treatment-A prospective, multicenter, randomized, double-blind, placebo-controlled clinical trial. Urol. Sci..

[19] Giannantoni A., Porena M., Costantini E., Zucchi A., Mearini L., Mearini E. (2008). Botulinum A toxin intravesical injection in patients with painful bladder syndrome: 1-year followup. J. Urol..

[20] Kuo H.C. (2013). Repeated onabotulinumtoxin-a injections provide better results than single injection in treatment of painful bladder syndrome. Pain Physician.

[21] Smith C.P., Radziszewski P., Borkowski A., Somogyi G.T., Boone T.B., Chancellor M.B. (2004). Botulinum toxin A has antinociceptive effects in treating interstitial cystitis. Urology.

[22] Jhang J.F., Kuo H.C. (2015). Novel treatment of chronic bladder pain syndrome and other pelvic pain disorders by onabotulinumtoxinA injection. Toxins.

[23] Gamper M., Viereck V., Eberhard J., Binder J., Moll C., Welter J., Moser R. (2013). Local immune response in bladder pain syndrome/interstitial cystitis ESSIC type 3C. Int. Urogynecol. J..

[24] Liu H.T., Shie J.H., Chen S.H., Wang Y.S., Kuo H.C. (2012). Differences in mast cell infiltration, E-cadherin, and zonula occludens-1 expression between patients with overactive bladder and interstitial cystitis/bladder pain syndrome. Urology.

[25] Schachar J.S., Evans R.J., Parks G.E., Zambon J., Badlani G., Walker S.J. (2019). Histological evidence supports low anesthetic bladder capacity as a marker of a bladder-centric disease subtype in interstitial cystitis/bladder pain syndrome. Int. Urogynecol. J..

[26] Jhang J.F., Hsu Y.H., Kuo H.C. (2013). Characteristics and electrocauterization of Hunner’s lesions associated with bladder pain syndrome. Urol. Sci..

[27] Homma Y. (2019). Interstitial cystitis, bladder pain syndrome, hypersensitive bladder, and interstitial cystitis/bladder pain syndrome—Clarification of definitions and relationships. Int. J. Urol..

[28] Chuang Y.C., Yoshimura N., Huang C.C., Chiang P.H., Chancellor M.B. (2004). Intravesical botulinum toxin A administration produces analgesia against acetic acid induced bladder pain response in rats. J. Urol..

[29] Liu H.T., Kuo H.C. (2007). Intravesical botulinum toxin A injections plus hydrodistension can reduce nerve growth factor production and control bladder pain in interstitial cystitis. Urology.

[30] Hanno P., Walsh P.C., Retik A.B., Vaughan E.D., Wein A.J. (1998). Interstitial cystitis and related diseases. Campbell’s Urology.

[31] Hanno P.M., Landis J.R., Matthews-Cook Y., Kusek J., Nyberg L. (1999). The diagnosis of interstitial cystitis revisited: Lessons learned from the National Institutes of Health Interstitial Cystitis Database study. J. Urol..

[32] Lubeck D.P., Whitmore K., Sant G.R., Alvarez-Horine S., Lai C. (2001). Psychometric validation of the OLeary-Sant interstitial cystitis symptom index in a clinical trial of pentosan polysulfate sodium. Urology.

[33] Abrams P., Cardozo L., Fall M., Griffiths D., Rosier P., Ulmsten U., van Kerrebroeck P., Victor A., Wein A., Standardisation Sub-committee of the International Continence Society (2002). The standardisation of terminology of lower urinary tract function: Report from the Standardisation Sub-committee of the International Continence Society. Neurourol. Urodyn..

[34] Hsiao S.M., Lin H.H., Kuo H.C. (2017). Videourodynamic Studies of Women with Voiding Dysfunction. Sci. Rep..

[35] Parsons C.L., Housley T., Schmidt J.D., Lebow D. (1994). Treatment of interstitial cystitis with intravesical heparin. Br. J. Urol..

